# Epidural haematoma: Rare complication after spinal while intending epidural anaesthesia with long-term follow-up after conservative treatment

**DOI:** 10.4103/0019-5049.76596

**Published:** 2011

**Authors:** Devalina Goswami, Jyotirmoy Das, Achyut Deuri, Ajit K Deka

**Affiliations:** Departments of Anaesthesiology and Critical Care, Gauhati Medical College, Guwahati, Assam, India

**Keywords:** Complications, epidural anaesthesia, epidural haematoma

## Abstract

Epidural anaesthesia (EA) is an extensively used procedure for many surgeries. Increase incidence of bleeding in the epidural space [epidural haematoma (EH)] is reportedly more common in patients with altered coagulation and patients on anticoagulation treatment. EH secondary to spinal while intending EA for caesarean section (C-section) in a healthy individual leading to transient or persistent neurological problems is very rare. We report a case of EH after spinal while intending EA for C-section in a healthy young female along with 5-yrs follow-up after conservative treatment.

## INTRODUCTION

Epidural anaesthesia (EA)[1,2] is being used widely and successfully as primary mode of anaesthesia or with general anaesthesia to alleviate per operative pain.[3] The technique offers many advantages, such as improved cardiopulmonary function, decreased incidence of deep vein thrombosis, less intraoperative anaesthetic, improved postoperative gut function, early tracheal extubation, better mobilization. Potential benefits of EA and analgesia are well reported; information about rare adverse events had not been reported. Epidural haematoma (EH)[1] is a very rare event following EA especially in patients without any risk factors.[2]

## CASE REPORT

A 25-year-old primipara without any anaesthetic risk or comorbidity had to undergo elective caesarean-section (C-section) for cephalo-pelvic disproportion. EA was planned for her. She was put in left lateral position and under all aseptic and antiseptic precautions an 18-G Tuohy needle was inserted at lumbar space 3-4. Loss of resistance was elicited with air but during the procedure dura was punctured inadvertently. Without removing the needle, 2.5ml of 0.5% bupivacaine heavy was injected to the sub-aracnoid space. Regional block was achieved up to Thoracic 6 level and C-section was completed uneventfully.

**Figure 1:**
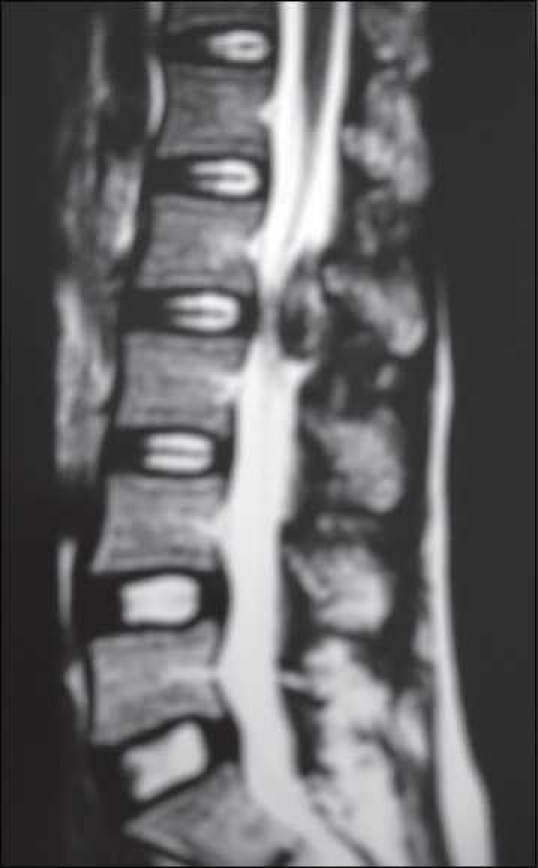
Magnetic resonance imaging picture showing the haematoma compressing the spinal canal

Patient was observed as routine in the postoperative ward. Initially she showed evidence of sensory regression after the block. However, after 8 hrs when leg movements were tried, patient was unable to lift her legs properly. She had diminished sensory reflexes and motor paresis. Power in lower limbs was 2/5 with definite inability to move limbs against gravity. Paresis was more pronounced on the left compared to the right side. Rest of her parameters were within normal limits. A para-spinal space occupying lesion was suspected and magnetic resonance imaging (MRI) was urgently requested. Owing to administrative and financial problems it could be done only after 24 hrs. MRI showed an EH at the site corresponding to the puncture [Figure 1]. As 24 hrs had already elapsed and patient and her relatives did not consented for high-risk decompression surgery she was managed conservatively with steroids and physiotherapy. Her coagulation profile was within normal limits during her stay in hospital.

Motor power gradually started improving over 7 days to 3/5 in both limbs against gravity and against mild resistance. On 15^th^ postoperative day she could stand with support; however, she had evidence of bilateral foot drop. On 60^th^ day follow-up her foot drop had improved substantially and she could walk with support. Patient was assured moral support and advised physiotherapy as the only care. On 180^th^ day follow-up she had fully recovered and did not show any evidence of residual disease. Since then she has been regularly being followed up every year and after 5-yrs follow-up she is leading a perfectly normal life.

## DISCUSSION

The incidence of EH is approximated to be less than 1 in 150,000 epidurals[4] and it’s even rarer following spinal anaesthesia (1 in 2,20,000)[5] in patients with no added morbidity. EH can lead to compression of the spinal nerves causing various degrees of irreversible damage.[6] Generally, patients with vertebral EHs complain of severe pain of abrupt onset in the spinal region and radicular pain, followed by sensory-motor deficit during the hours or days following the beginning of the disease. The actual incidence of neurological dysfunction resulting from haemorrhagic complications is unknown.[5]

Cases of EH have been reported to occur in patients who receive anti-coagulants, have bleeding disorders, or after traumatic needle insertion. Approximately 50% of EHs are associated with catheter removal.[7–9] As many as 13% of cases with EH are reportedly seen in patients without any preoperative risk factors.[8]

Results concerning postoperative morbidity with per operative epidural analgesia have been contradictory. A recent review[10] of intrathecal and EA and analgesia for cardiac surgery, suggests that while they provide enhanced postoperative analgesia, a “clinically important effect on morbidity and mortality” has not yet been demonstrated. Favourable outcomes are described for EH in adolescent patients.[11] Patients with hemi paresis have often been misdiagnosed as cerebral infarction.[2] There are many factors that may predispose to “spontaneous” EH. Pregnancy, physical strain resulting in an increase in epidural plexus venous pressure, arterial hypertension and vasculitis, especially systemic lupus erythematosus are factors that can lead to spontaneous EH.[12] Among other theories a venous origin is often assumed for spinal epidural haemorrhage. In our case the needle probably touched a pregnancy related dilated epidural vein causing trauma.

MRI is the most preferred investigation as it can rapidly confirm the diagnosis of EH as well as provide the accurate estimate of size, location and extension.[12] MRI also allows inspection of the segmental nerve roots, the spinal cord and its membranes.

An unpredictable extensive EH can occur after any trauma to spine in patients at low risk for haemorrhage. Spontaneous spinal EHs require a prompt diagnosis because neurological prognosis essentially depends on the interval of time between onset of symptoms and surgical decompression. A significant period of the ‘golden hour’ of intervention is usually lost, because of the residual effect of the local anaesthetic and lack of proper monitoring. Nerve injury may be minimized by surgical decompression within 24 hrs of the first symptoms.[13] This case illustrates that immediate surgical intervention may not always be necessary in certain patients with early recovery.

## CONCLUSIONS

A high degree of suspicion is a must for any neurological deficit in the recovery period after neuroaxial intervention even in unsuspecting patients. Conservative management with careful observation may play a role as a management option for patients with EH presenting with neurological dysfunction if neurological recovery is early and sustained.
